# The social paediatrics initiative: a RICHER model of primary health care for at risk children and their families

**DOI:** 10.1186/1471-2431-12-158

**Published:** 2012-10-04

**Authors:** Sabrina T Wong, M Judith Lynam, Koushambhi B Khan, Lorine Scott, Christine Loock

**Affiliations:** 1 University of British Columbia, School of Nursing and Research, 6190 Agronomy Road, #302, Vancouver, V6T 1Z3, BC, Canada; 2 University of British Columbia, Centre for Health Services Policy, #201-2206 East Mall, Vancouver, V6T-1Z3, BC, Canada; 3 Children’s Hospital, K1-111, 4480 Oak Street, Vancouver, V6H 3V4, BC, Canada; 4 Sunny Hill Health Centre for Children, 3644 Slocan Street, Vancouver, V5M 3E8, BC, Canada; 5 Department of Paediatrics, UBC Faculty of Medicine, 4480 Oak Street, Vancouver, V6H 3V4, BC, Canada

**Keywords:** Primary care, Public health, Vulnerable populations, Innovative model, Empowerment, Community engagement

## Abstract

**Background:**

The Responsive Interdisciplinary Child-Community Health Education and Research (RICHER) initiative is an intersectoral and interdisciplinary community outreach primary health care (PHC) model. It is being undertaken in partnership with community based organizations in order to address identified gaps in the continuum of health services delivery for ‘at risk’ children and their families. As part of a larger study, this paper reports on whether the RICHER initiative is associated with increased: 1) access to health care for children and families with multiple forms of disadvantage and 2) patient-reported empowerment. This study provides the first examination of a model of delivering PHC, using a Social Paediatrics approach.

**Methods:**

This was a mixed-methods study, using quantitative and qualitative approaches; it was undertaken in partnership with the community, both organizations and individual providers. Descriptive statistics, including logistic regression of patient survey data (n=86) and thematic analyses of patient interview data (n=7) were analyzed to examine the association between patient experiences with the RICHER initiative and parent-reported empowerment.

**Results:**

Respondents found communication with the provider clear, that the provider explained any test results in a way they could understand, and that the provider was compassionate and respectful. Analysis of the survey and in-depth interview data provide evidence that interpersonal communication, particularly the provider’s interpersonal style (e.g., being treated as an equal), was very important. Even after controlling for parents’ education and ethnicity, the provider’s interpersonal style remained positively associated with parent-reported empowerment (p<0.01).

**Conclusions:**

This model of PHC delivery is unique in its purposeful and required partnerships between health care providers and community members. This study provides beginning evidence that RICHER can better meet the health and health care needs of people, especially those who are vulnerable due to multiple intersecting social determinants of health. Positive interpersonal communication from providers can play a key role in facilitating situations where individuals have an opportunity to experience success in managing their and their family’s health.

## Background

Health inequities refer to potentially remedial differences in health or access to care that can result from structural arrangements; in this sense, inequities may be deemed unjust [1]. Indeed, population analyses suggests that health inequalities are magnified due to social determinants of health which can lead to inequitable access to health care [2]. Barriers in accessing care can lead to inequities in health for both adults and children. Even in a country such as Canada that has a universally accessible health care system, obtaining needed health care from a regular source of care varies provincially; approximately 75% of Quebec residents, 88% of British Columbia residents to 93% of Nova Scotia residents report having a family physician. Although many residents have difficulties in accessing care, families and children who are made vulnerable by multiple intersecting social determinants of health face both social and structural challenges in accessing health care.

Inequities in health have become a central concern of health services delivery and health policy in Canada and countries throughout the world. The World Health Organization (WHO) [4] reports that one of the most efficient ways of “closing the gap” in health inequities within a population is to address the needs of those who are most disadvantaged. One group who are most disadvantaged are children living in poverty who are often at higher risk for developmental delay or poor physical or mental health [5,6]. They often miss out on routine screening and do not benefit from diagnostic assessment, treatment and/or early intervention and are at higher risk for needing services from multiple sectors and/or through a number of developmental stages. When primary health care (PHC) is not accessible or effective, their health may worsen, there is more reliance on emergency care, and they lose the benefits of continuity of care [7,8]. This lack of early recognition and assessment are recognized as posing the greatest threat to child health and wellbeing and as having the greatest negative impact on children’s health trajectories [9,10]. Moreover, past work suggests that families with multiple forms of disadvantage (e.g. poverty, English as a second language) cannot easily navigate the complexities of the health care system, nor always enact recommended treatments without additional support [11,12]. It is particularly concerning that the health impact of social and structural forms of disadvantage are associated with poor health are cumulative over the life course [13,14].

In 2005 researchers mapped the development of all children in British Columbia at school entry. The developmental mapping initiative identified that along with behavioral and learning problems 67.2% of children living in Vancouver’s inner city entered kindergarten delayed in communication and general knowledge skills [15]. In addition to these developmental profiles, 35% of children in this neighbourhood live in single-parent headed households and 44% speak English as a second language [15]. One in five children has parents who are immigrants, refugees or who are Aboriginal. Each of these characteristics has been associated with children’s increased ‘risk’ for poor health or development.

The Responsive Interdisciplinary Child-Coordinated Community Health Education and Research (RICHER) initiative is an intersectoral and interdisciplinary community outreach PHC model. It is being undertaken in partnership with community based organizations in order to address identified gaps in the continuum of health services delivery for ‘at risk’ children and their families. A significant number of children were identified as ‘at risk’ because of their developmental profiles and their social circumstances. The RICHER initiative seeks to dismantle the structural and social barriers that limit access to, and provision of, needed primary and specialty health care services for inner city children who are ‘at risk’.

The RICHER initiative has two main goals. First, it aims to increase access to needed primary health care, public health, and specialist services for children living in Vancouver’s inner city, who are vulnerable because of their social or material circumstances. Second, it aims to empower parents of such children to become more active participants in the care of themselves and their children by acquiring knowledge of their child’s health condition, knowledge of the resources available to manage health conditions, and awareness of how to access such resources. As part of a larger study, this paper reports on whether the RICHER initiative is associated with increased:

1) Access to health care for children and families with multiple forms of disadvantage; and

2) Parent-reported empowerment

### Social paediatrics and the RICHER initiative

The RICHER initiative, informed by social paediatrics, is an intervention that provides access to PHC services and, referral for specialized assessments or treatment, while also considering the social conditions that contribute to ‘vulnerabilities’. Social Paediatrics is a philosophy and an approach to practice that locates the child at the centre of care [16,17]. Providers using a social paediatrics approach report that it effectively addresses the health needs (e.g. developmental and emotional challenges) of children who are disadvantaged because of intersecting determinants of health and having been victims of violence or abuse. The RICHER initiative uses a social paediatrics approach [16] to provide services in the child’s neighbourhood and, in our case, is undertaken in partnership with community resources; the importance of enduring socially supportive relationships can mitigate the risk of health inequities for vulnerable children [18] and, it is founded on a belief in the competence of children, parents and families. In this case it is a ‘best practice approach’ intended to address inequities in child health. It is informed by insights from the foundational work on social paediatrics as articulated by Julien [16]; by related research and practice in community paediatrics [19-21] and by Lynam and colleagues’ programs of research with marginalized groups [22-25].

Social paediatrics and the RICHER initiative have three central premises. First, the social paediatrics approach is premised on the assumption that the nature of relationships built between the health care providers and the child and his/her family are key to ensuring continuity in service delivery and to ensuring that the parents are supported and equipped to nurture their child’s development. In enacting this approach providers seek to create avenues for connection, taking into account the child’s strengths and abilities in order to tailor care and communication in ways that foster the child’s development [17,26]. The social paediatrics approach is not only an innovative model of PHC service delivery, it serves as a model for establishing relationships with vulnerable children and their families to meet their health and developmental needs.

Second, the social pediatric approach is located in the child’s community. Providers use interventions that work to strengthen the relationships with providers but also to build networks with people who offer a range and diversity of community-based resources for children and their families. The approach seeks to be responsive by tailoring interventions to be timely, supportive, and effective in working with the target population [24,25]. The RICHER initiative purposefully partnered with community-based organizations to foster access and extend the types of resources and supports available including access to resources to address social determinants of health; past work suggests that building relationships through intersectoral collaborations for a common goal can buffer the negative effects of material disadvantage [27-30].

Finally, the social paediatrics approach and the RICHER initiative seek to empower both its patients and their families to care for their health and well-being. Individual families are empowered to care for their health. Empowerment is defined as a process by which people gain mastery over their lives [31]. Providers working in the RICHER initiative try to empower their patients, by supporting them, guiding them to acquire knowledge of their own and their child’s health condition or developmental stage, offering strategies for managing their child’s health condition and connecting them with resources. Such action encourages patients to assume personal responsibility for their health and imparts the idea that what patients do influences their health [32]. In the case of RICHER, the providers actively work with primary caregivers, families and key people in their networks of support.

## Methods

### Design and participants

This was a mixed-methods study that gathered both quantitative and qualitative data; it was undertaken in partnership with the community, both organizations and individual providers. In an effort to undertake research that is respectful of peoples’ histories, and to address questions identified as priorities within the community the broader research study was designed using participatory methods [33]. A critical participatory approach to inquiry emphasizes collaboration and reflection and is marked by features such as reciprocity, inter-subjectivity, reflexivity, and the co-construction of knowledge [34]; insights are generated by recognizing and reconciling multiple viewpoints. As the study context is a community with an, often negative, history of ‘surveillance’ by researchers and practitioners, it was particularly important that the research approaches accorded recognition to multiple voices and developed strategies to gain an understanding of the perspectives of those who have been traditionally overlooked in health research. The organizations, providers, and research team worked together to articulate the principles of practice and research, identify successful data collection methods (both format and recruitment procedures), and interpretation of the findings.

The site where this study took place was in one of Canada’s poorest areas, adjacent to Vancouver’s downtown eastside. Most, if not all residents of this area face multiple forms of disadvantage, including poverty, living in sub-standard or having no housing, speaking English as a second language, a history of mental health problems, and repeated exposure to trauma or violence. A convenience sample of eligible participants for this component of the study were English or Chinese-speaking (Cantonese dialect) families who had used the RICHER primary care services at least one time in the last 12 months. The person in the household that was interviewed, on behalf of the child, was his/her main caregiver. Given that the providers deliver many primary care services in community spaces such as community centres and day cares, families were recruited for this study in these areas. Recruitment was also facilitated through the distribution of flyers to the community spaces and posters in the clinic. Participants received a $15 honorarium in appreciation for their time.

In this paper we report on the data gathered using a standardized questionnaire, and unstructured qualitative interviews undertaken to engage in a more full exploration of individual participants’ responses.

### Procedures

A combination of face-to-face surveys and in-depth interviews were completed in either English or Cantonese. All interviews were conducted over six months: February – August 2010. The survey reflected important dimensions of PHC (e.g. strength of affiliation, accessibility, continuity (informational, relationship and management continuity), interpersonal communication, patient activation) based on our previous work [32,35], sociodemographic characteristics, and health status, and confidence in the health care system. The dimensions of PHC were measured using a combination of items and scales. All scales and items measuring dimensions of PHC were publicly available, except for “NP Knowledge of Child”. If items were part of a scale, internal consistency reliability was examined using Cronbach’s alpha coefficient. Construct validity was assessed by examining whether the scales correlated as expected with related scales. Scales had adequate internal consistency reliability. The Interpersonal Processes of Care scales [31], consisting of three dimensions (communication, interpersonal style, and shared decision-making), had a Cronbach alpha ranging from 0.64 to 0.76. The NP Knowledge of Child scale had a Cronbach’s alpha of 0.84. All interpersonal scales have shown adequate construct validity where they were more highly correlated with each other than to other scales [35]. All items and scales have undergone a rigorous forward-backward translation to ensure semantic equivalence [36,37]. A convenience sample of parent/caregivers of children who had attended the clinic completed the surveys.

Purposive sampling was used to identify interviewees for the qualitative interviews. Based on answers provided for the survey, potential participants for the in-depth interviews were asked to participate. Interviewees also participated because they wanted to expand upon an answer they provided for the survey. For example, interviewees were asked to elaborate on their responses regarding their involvement in decision making, how they perceived their care to be coordinated, and to provide examples of how their primary care experiences through RICHER compared to other primary care clinics they had used. In addition, field note data were collected from interviewers’ observations during both the survey and in-depth interviews [38]. All in-depth interviews were audio-recorded, where pertinent translated, and transcribed. All procedures were approved by our community partners, the University British Columbia ethics board and other jurisdictional review boards.

### Data analysis

Data analysis involved a two-staged process. First, we analyzed the data using standard quantitative or qualitative techniques. Univariate and logistic regression analysis of the survey data allowed for an examination of primary care experiences associated with patient empowerment. Our independent variables of interest included different dimensions of primary care (see Table 1): strength of affiliation [13,39] with a usual source of care, and interpersonal communication (communication, shared decision-making, and provider’s interpersonal style) [40]. Strength of affiliation is an index based on a series of questions about the place, doctor or Nurse Practitioner that knows the child best, that is most responsible for the child’s health, and that the parent usually takes the child to when the child is sick. For the purposes of this study, interpersonal communication consisted of four scales, Clarity of Communication (In the past 12 months, “*How often did your nurse practitioner speak too fast?”, “How often did your nurse practitioner use words that were hard to understand?”)*, Explained Results (In the past 12 months, (In the past 12 months, *“How often did your Nurse Practitioner clearly explain your child’s test results such as X-rays, blood tests or developmental screening tests?”, “How often did your Nurse Practitioner clearly explain results of your child’s physical exam?”),* Shared Decision-Making *(*In the past 12 months, “*How often did you and your nurse practitioner work out a treatment plan together?”, “If there were treatment choices, how often did your nurse practitioner ask if you would like to help decide your treatment?”),* and Interpersonal Style *(*In the past 12 months, “*How often was your nurse practitioner concerned about your feelings?”, “How often did your nurse practitioner really respect your child?”, “How often did your nurse practitioner doctor treat you as an equal?”).* Responses to these items ranged from always, usually, sometimes, rarely, and never. The NP knowledge of the child consisted of three items: *“How would you rate your Nurse Practitioner’s knowledge of your child’s entire medical history?”, “How would you rate your Nurse Practitioner’s knowledge of what worries you most about your child’s health?”, and “How would you rate your Nurse Practitioner’s knowledge of your responsibilities at home, work or school?”.* Responses for the NP knowledge ranged from excellent, very good, good, fair, and poor. 

**Table 1:** Experiences of primary care

**Characteristics**	**Distribution or mean (N=86)**
**Strength of Affiliation:**	
Strength of Affiliation, grouped (%)	
No/Weakest affiliation	11.6
Weak/Less Strong affiliation	24.4
Strong affiliation	64.0
**Number of times used clinic in past 12 months,** M (SD)	7.2 (10.5)
**First Contact Accessibility**	
How quickly is your child able to see the nurse practitioner when the appointment you need is for common health problems? (%)	
The same day	61.6
The next working day	5.8
Within 3 or more working days	27.9
How quickly has your child been able to see the Nurse Practitioner when the appointment you need is for an urgent, but minor health problem? (%)	
The same day	66.3
The next working day	11.6
Within 3 or more working days	10.4
How long do you and your child usually have to wait at your Nurse Practitioner’s office or place of care from the time of your appointment until your visit begins? (%)	
0–10 minutes	61.6
11–20 minutes	22.1
21–30 minutes	10.5
31+ minutes	4.7
**Experience with Social Paediatrics Initiative (SPI) Clinic**	
In the past 12 months, has your child’s development been assessed? (%)	
Yes	54.7
Among those assessed, concerns identified: (%)	
Speech and Language	23.4
Learning	19.1
Social/emotional development	17.0
Behavioral problems	14.9
Other:	
Physical ability	8.5
Vision	4.3
Hearing	6.4
Among those who had been assessed, was child referred to:	
Development services	19.8
Supported child care or having special needs	20.9
SPI provider/team participated in, or helped you to organize a meeting to discuss about your child and put in place a plan of care with others? (%)	
Yes	27.9
**Relationship Continuity**	
See the same provider? (%)	
Always	51.2
Usually	30.2
Sometimes	9.3
Rarely/Never	8.2
^2^NP knowledge of child (3 items)	
M(SD)^3^	3.9 (1.0)
Observed range	1 – 5
**Cultural Responsiveness**	
Difficulty getting health care because your cultural ways were not taken into consideration? (%)	
Never	46.5
Rarely	8.1
Sometimes	8.1
Usually/Always	1.2
Language barriers when trying to get the ongoing care that you or your child needed? (%)	
Never	36.0
Rarely	10.5
Sometimes	11.6
Usually/Always	5.8
**Communication**	
^1^Interpersonal Processes of Care (IPC) General Clarity of Communication scale (2 items)	
*M (SD)*	4.6 (0.6)
Observed range:	3 – 5
^1^IPC Explained Results scale (2 items)	
*M (SD)*^*4*^	4.3 (0.9)
Observed range	2 – 5
**Decision Making**	
^1^IPC Decision Making scale (2 items)	
*M (SD)*^5^	3.7 (1.3)
*Observed range:*	1 – 5
How important is it to you to have a Nurse Practitioner include you in the decision-making of treatment plans for your child?	
Not important at all	3.5
Somewhat important	16.3
Very important	77.9
**Interpersonal Style**	
^1^IPC Compassionate, Respectful scale (3 items)	
*M (SD)*^6^	4.7 (0.5)
Observed range:	2.7 – 5
How important is it to you to have a Nurse Practitioner that treats you as an equal?	
Not important at all	0.0
Somewhat important	10.5
Very important	88.4
**Patient Empowerment**	
^1^Empowerment Scale (6-items)	
*M (SD)*^7^	4.2 (0.9)
*Observed range:*	2 – 5

^1^Potential range of scales are 1–5 where a higher score equals more of the concept; 5-always, 4-usually, 3-sometimes, 2-rarely, and 1-never. ^2^Potential range of scale was 1–5 where a higher score equals more of the concept; 5-excellent, 4-very good, 3-good, 2-fair, 1-poor. ^3^N=2 missing (2.3%), ^4^N=42 missing (48.8%) because items in the scale were not applicable, ^5^N=34 missing (39.5%) because items in the scale were not applicable, ^6^N=1 missing (1.2%), ^7^N=10 missing (11.6%).

Note. Frequencies may not add to 100% due to missing data because items were not applicable.

Our dependent variable of interest was patient empowerment. Empowerment [40] is a six-item likert scale that asked about how often the SPI provider encouraged the parent in regard to the child’s health or encouraged the child about his/her health. For ease of interpretation, the Empowerment scale was dichotomized into never/rarely/sometimes (0) and usually/always (1) for the logistic regression. The items in the empowerment scale were: “*How often did your Nurse Practitioner: praise you for how you were taking care of your child’s health? help your child feel that sticking with the treatment would make a difference? help your child feel that his/her everyday activities such as diet and lifestyle make a difference in his/her health? help you feel confident about your ability to take care of your child’s health? help you feel that you have a sense of control over your child’s health? help you feel like you can prevent some of your child’s health problems?”* For all scales, a score was only calculated if at least 60% of the items were answered. All scale scores ranged from 1–5 where a higher score indicated more of the construct.

In-depth interview/field note data were analyzed using thematic analysis according to procedures for qualitative data [41]. Interview transcripts were repeatedly read by the research team to identify recurring and contradictory patterns in the data, and links to theoretical work. A coding scheme was developed for the patient and health care provider data. NVivo, a qualitative software package, was used to organize the data. Quality of the data was ensured by attention to the depth and variation of the data gathered [41]. Credibility of the analysis was continually evaluated with the research team, which included members of the community organizations and RICHER providers.

Second, we performed mixed methods data analyses in order to a) seek convergence and corroboration of results from the different methods and b) achieve complementarity where the interview results enhanced and clarified the survey results [42]. For example, the analysis of survey data and the thematic analysis of interview data allowed us to compare the participants’ ratings of ‘access’ to those identified in past research and also gain a more comprehensive understanding of the complexities of ‘access’ and the role of relationships in fostering access. We gave equal priority to quantitative and qualitative data and concurrently analyzed the survey and interview/field note data. Quotes from qualitative data seen in the results section are each from different parents. We present the mixed-method analysis in the results section.

## Results

### Description of the sample

A total of 86 respondents completed the survey about their child’s PHC experiences and 8% also completed an in-depth interview. Table 2 shows the characteristics of the parents, most of whom were mothers. The mean age was 37.7 years with over half (54%) being single parent households and reporting their highest level of education was grade 12 or lower (50%). Almost two-thirds of the respondents reported they were either of Chinese (33%) or Aboriginal (29%) heritage. While English was the predominant language spoken at home, half of the respondents reported being immigrants to Canada (51%). Fewer than one quarter of the respondents (19%) worked full time and almost half worked either part-time (15%) or reported they were looking after their home/family (33%). Thirteen percent of parents reported they were unable to work due to long-term illness or disability. The majority of household incomes were low with 41% of respondents reporting an income range of $10,000-$30,000. Most respondents were renting (81%) and many (43%) reported receiving financial support for housing.

**Table 2:** Characteristics of the sample

**Characteristics of parent who answered the survey:**	**Distribution or mean (N=86)**
Gender of parent (%)	
Female	91.9
Age of parent, *M (SD)*	37.7 (9.4)
Single parent (%)	
Yes	53.5
Marital status (%)	
Married or living with a partner	47.7
Separated/Divorced/Widowed	23.3
Never Married	24.4
Highest level of education (%)	
Did not complete secondary school or high school	26.7
Completed secondary school or high school	23.3
Had some university education or completed a community college, technical college, or postsecondary program	30.2
Completed a bachelor’s, graduate or professional degree	16.3
Work status (%)	
Employed^1^:	
Full-time	18.6
Part time	15.1
Unemployed/looking for work	12.8
Looking after your home/family	32.6
At school/full-time education^2^	3.5
Unable to work due to a long-term sickness or disability	12.8
Retired/other	2.3
Ethnicity (%)	
White	23.3
Chinese	32.6
Aboriginal (First Nations/Metis)	29.1
Other (Korean, Latino, Filipino, African, mixed ethnicity)	15.1
Born in Canada (%)	
No	51.2
For those not born in Canada, # of years living in Canada, *M(SD)*	11.0 (7.6)
Range	0–31 years
Language spoken at home (%)	
English	58.1
Cantonese/Mandarin	32.6
Other	9.3
Total household size (%)	
1–2	22.1
3	19.8
4	25.6
≥5	32.6
Number of children < 18 years old in household (%)	
0	4.7
1	39.5
2	29.1
3	14.0
4 or more	12.8
Household income (%)	
Less than $10,000	25.6
$10,000 - $30,000	40.7
$30,000 - $50,000	11.6
$50,000 - $80,000	5.8
More than $80,000	3.5
Type of housing (%)	
Apartment	50.0
House	44.2
Other (e.g. room, basement suite)	5.8
Length of time in home, *M (SD)*	3.8 (3.3) years
Range	½ month - 15 years
Rent or own? (%)	
Rent	81.4
Own	14.0
Other	2.3
Receives financial support for housing (%)	
Yes	43.0
**Characteristics of the child that the parent focused on in the Survey:**	**Distribution or Mean (N=86)**
Overall general health (%)	
Excellent/Very good	51.2
Good	31.4
Fair/Poor	17.4
Diagnosis of long-term or chronic health problems (%)	
Yes	23.3
For those with long-term or chronic health problem(s), number of health problems, M(SD)	1.5 (0.7)
For those with long-term or chronic health problem(s) (%):	
Developmental (e.g. ADHD, autism, delayed speech)	45.0
Congenital (e.g. Fetal Alcohol Syndrome)	50.0

^1^This refers to employment outside of the home. It also includes self-employment and work training programs.

^2^Includes one parent who gave two answers: ‘at school/in full-time education’ and ‘unemployed/looking for work’.

Note. Frequencies may not add to 100% due to missing data. There was too much missing data to calculate mean age of children. Sex of child was not asked.

Most respondents reported living in a household with 4 or more people (57%) where there were one or two children. About one-quarter of respondents (27%) reported living in a household where there were three or more children. The majority of children (82%) attending RICHER were reported to be in excellent, very good, or good health. Almost one-quarter of the children (23%) had been diagnosed with a chronic health problem, almost equally represented by congenital and developmental concerns (e.g. birth defects, fetal alcohol syndrome), autism, and speech and language delay.

### Parents’ reports of their experiences with RICHER

Table 1 shows the survey respondents’ experiences using RICHER. Parents reported using the clinic an average of seven times in the last year where 64% reported having a strong affiliation with their usual source of care. Seventy-eight percent of respondents report being able to access RICHER either the same day or the next working day for urgent, but minor health problems (e.g. skin rash, bed bugs) and 66% report being able to see a RICHER provider within this same time frame for common health problems (e.g. cold and cough). The majority (84%) of respondents reported waiting 20 minutes or less for their scheduled appointment time. Continuity of care was provided by seeing the same provider for most respondents (81%) and having the provider involved in coordination of care (28%) between RICHER, public health, and specialist care, when needed. Importantly, parents reported that the RICHER providers identified one in every five children needing further support, including access to specialized services, from the health care system for developmental delays in: speech and language (23%), learning (19%), social/emotional (17%), behaviour (15%), other areas such as vision and hearing.

The majority of families who reported a child with chronic condition were coping with a developmental or a congenital condition (e.g. congenital heart disease, cerebral palsy, ADHD, autism spectrum disorder, Fetal Alcohol Spectrum Disorder) that influenced their child’s development. The survey data collected from parents asked that they only report on one of their children who used RICHER. The interview data provide more contextual detail; importantly families actually had multiple children with a long-term or chronic condition.

The following data excerpt enhances the survey results by illuminating health inequities are addressed using RICHER. Thematic analysis suggests that RICER provided opportunities for increased access to specialty care and coordination of care across the system. This parent discusses how the RICHER initiative increased access to the full spectrum of health care services. In this case the primary care provider has worked with the child in his environment, has assisted the child by fostering access into specialized services, and has worked with the parent to foster her understanding of, and her ability to act on, the assessment information.

"“Parent: Oh, walk in clinics I’ve been there many times, but it’s a nightmare…because there are a lot of people. I have a problem because my little one is ADHD. He’s really hyperactive and then when I come in, it’s really difficult…."

"Interviewer: In your experience have you found that (the nurse practitioner) has responded to your concerns? Across all the visits or maybe could you give an example from one of the visits?"

"Parent: Umm, she just works, as a part of the team with the daycare, and to uh, to my son get his pediatrician… she just worked hard to try and get my son’s (developmental) assessment to (tertiary centre)……. He needed [a] pediatrician, he needed [an] assessment, you know…Before him, I was a mother to 3 kids, but I never had a little one….he is really, really energetic………For me, it was (one of the NPs). She talked to daycare and phoned to social worker and….all of them I know are working (together).….It’s good, you know to have someone interested in your kid’s problem because sometimes you don’t know what to do. You aren’t a professional. You don’t know what they need.”"

This parent felt that she gained support for dealing with a number of health challenges for one of her children and recognized the need for a referral to mental health services for a 2^nd^ child. In this case the mother identified she needed more than the episodic care provided at the walk-in clinics she had accessed in the past. She also recognized the limits of their own expertise (e.g. she had limited knowledge of child development, limited knowledge of ways to manage her children’s health conditions, and limited knowledge of how to navigate the health care system to access appropriate health care services).

The survey data suggest most respondents did not have language barriers (58%) in accessing RICHER. Even though many families reported speaking languages other than English (and many were immigrant families), they reported high scores on communication with the provider and the provider’s interpersonal style. Respondents found the communication clear, that the provider explained any test results in a way they could understand, and that the provider was compassionate and respectful. Lower scores were reported for shared decision-making. Over three-quarters of respondents reported that both shared decision-making (78%) and provider’s interpersonal style (88%) (e.g. being treated as an equal) was very important. Respondents scored high on the patient empowerment scale.

Another theme across the interview data was the importance of interpersonal communication between parents and their child’s primary care provider. This next quote illustrates the importance of being treated with respect. This parent has four children, one of whom has mobility, speech language, neurological and developmental challenges.

"“Interviewer: In the past 6 months have you ever experienced difficulty getting health information at this (RICHER) clinic?"

"Parent: No."

"Interviewer: What about at your (former) family doctor?"

"Parent: Yes. The doctors, sometimes the nurses (at the doctor’s office) aren’t very nice. Sometimes the doctor’s like arrogant, prideful. Very arrogant like….Sometimes when you ask the nurse something, they’re like arrogant. They say you have to wait."

"Interviewer: Like they rush you?"

"Parent: Yes."

"Interviewer: Have you experienced this at this (RICHER) clinic?"

"Parent: No."

"Interviewer: Okay. When you come to this clinic, do you feel that (the provider) respected you and your children?"

"Parent: Yes…….She’s very nice. Anything she does she asks you first for your opinion. The children also like her a lot. She usually first talk to the children, play with them a little first.”"

This parent also emphasized the respectful treatment (of herself and her children) and the benefits of having the provider help her understand her health and her children’s health challenges.

Taken together, the survey and interview data provide evidence that interpersonal communication, particularly respect, is related to parent-reported empowerment. The survey data, the univariate and logistic regression results are shown in Table 3. Reports of increased interpersonal processes of care communication and interpersonal style and NP knowledge of the child were significantly related to empowerment. The univariate results also suggest that our variables of interest, except for Strength of Affiliation with a usual source of care, were significantly related to empowerment. However, the logistic regression model suggests that after controlling for parents’ education and ethnicity, only the provider’s interpersonal style remains positively associated with patient empowerment (p<0.01). This result suggests that the relationship with the provider could play an important role in empowering parents who use RICHER.

**Table 3:** Univariate and logistic regression models for primary care experiences and patient empowerment (N=86)

			**Univariate**		**Demographics & experience logistic regression (R**^**2**^**= 0.47)**	
**Variable**	**N**	**%**	**Odds ratio**	**Overall p value/CI**	**Odds ratio**	**Overall p value/CI**
Education				**<0.05**		NS
Did not complete high school or secondary school	22	30.6	**0.14**	**(0.04, 0.55)**	0.29	(0.04, 2.26)
Completed high school or secondary school	18	25.0	0.37	(0.09, 1.62)	1.04	(0.08, 13.49)
At least some college, university or post-secondary	32	44.4	(ref)		(ref)	
Self-reported Ethnicity				**<0.01**		NS
White/Caucasian/European	15	20.8	(ref)		(ref)	
Chinese	24	33.3	**0.13**	**(0.02, 0.71)**	0.98	(0.06, 15.75)
Aboriginal (First Nations/Metis)	21	29.2	1.46	(0.18, 11.74)	6.09	(0.21, 174.05)
Other	12	16.7	0.46	(0.06, 3.35)	0.92	(0.05, 18.59)
Strength of Affiliation				NS	not included in model	
Scale range 0 to 4, treated as continuous			0.64	(0.35, 1.20)		
IPC Communication: Clarity of Communication				**<0.005**		NS
Scale range 3 to 5, treated as continuous			**3.94**	**(1.64, 9.45)**	1.65	(0.46, 5.94)
IPC Style: compassionate, respectful				**<0.0001**		**<0.01**
Scale range 2.67 to 5, treated as continuous			**34.52**	**(7.54, 157.93)**	**20.08**	**(3.04, 132.59)**
NP Knowledge of child				**<0.001**		NS
Scale range 1.67 to 5, treated as continuous			**3.46**	**(1.72, 6.99)**	2.43	(0.79, 7.44)

Note. Bold indicates statistically significant results. Empowerment was the dependent variable where “never/rarely/sometimes=0, usually/always=1”. NS=not significant. IPC communication scales: Elicited Concerns and Shared Decision-making were not included in these models due to high amounts of missing.

The last theme found across the interview data was the importance of relationships that parents have with the health care providers. In this case the children are coping with their own developmental challenges, a situation that is compounded by the fact that the parent is struggling with mental health issues and recently was injured in an accident. The mother notes that these health challenges are also compounded by her immigrant status, lack of fluency in English and her limited income. She reports often feeling misunderstood and feels alone due to separation from friends and family. She relies upon community centre resources for support and to assist her to ensure she has adequate food for her family. She also indicates that coping with these multiple challenges has magnified her feelings of not being able to provide for her children. As she explains, she frequently sought help for her own health challenges through emergency services.

"“Parent: And all the people that I called too weren’t there and I didn’t want to call the crisis line because they always misunderstand me in terms of asking for help. They think that, I do want to hurt myself but sometimes I just want to talk to someone. Because like when you have other languages, when you speak other language maybe you say something that it means something else, but they don’t understand.”"

"The interpersonal style of the provider was important in narrowing the gap in health inequalities since the provider was able to connect this woman with the resources she needed,"

"“Interviewer: Right. What about the (community centre) team?"

"Parent: Yeah, they, the (community centre) team they help me with food, with counselling in terms of my older son, my teenager, they talk to him. They help the little one by putting him in a program here for, that he works, you know, they call, and they help me, talking to me and refer me to places too. And they were the ones who refer me to the nurses here, so.”"

For these parents, having someone who cares about them and who knows is knowledgeable of the range of influences on their child and family situations is highly valuable.

## Discussion

The analyses of these data suggest that the RICHER initiative can provide access to care across the continuum of health care services for children (and their families) who are vulnerable because of their social and material circumstances. Health inequities often seen in those who cannot access health care could decrease with the implementation of RICHER. These results provide the first examination of an innovative model of delivering PHC, using a Social Paediatrics approach. Respondents were disproportionately poor, had lower education than the provincial average, and many had a child or children who had an identified developmental delay or chronic health condition. This model of PHC delivery is unique in its partnerships among health care providers and community members. RICHER has also developed a number of mechanisms for community engagement and public participation in order to ensure that there is both community input about services and accountability on service delivery to the community [33].

These results also provide additional evidence that the interaction between the provider and patient is an important factor associated with the patient reported outcome measure of empowerment. The provider’s interpersonal style of compassion and respectfulness was key to empowering patients to care for their health and the health of their children. This type of interpersonal style likely improves the provider’s and patient’s perceptions of trust in each other. Mutual trust improves cooperation, reduces the need for monitoring [43] and, in adults is associated with more appropriate prescribing of opioid analgesics [44]. Past work demonstrates the importance of mutual trust [45].

Interpersonal communication has been identified as an important process of care in achieving desirable outcomes of PHC, particularly for those at high risk of premature mortality or morbidity. In this case, children living in the inner city shoulder a disproportionate burden of illness and their health challenges are compounded by the multiple vulnerabilities and many social determinants of health*.* Yet, past evidence suggests that individualized interventions matched with an individual child's needs can change a poor health trajectory [46,47].

The RICHER initiative is an innovative model of PHC delivery that: a) facilitates access to programs that affect other social determinants of health such as housing and support networks, b) delivers any needed services through an interprofessional team that includes nurse practitioners and pediatric specialists, and c) is, in part, accountable to the community partners and engagement process. Patients, parents and their children, who use this initiative are empowered to manage their health and the health of their family because they learn how to navigate the health care system because of their interactions with the RICHER providers and use the network of supportive services operated by the community agencies. Given the level of concern for vulnerable populations, and that the possibility that poor health and developmental outcomes can be averted, the need for support in using appropriate resources as well as timely referral, diagnosis, treatment, has been underscored in past work [48].

The study is not without limitations. Although this sample size captured almost all families using the RICHER initiative at the time the data were being gathered, its generalizability is limited given the small sample size of the survey respondents. Additionally, some questions did contain missing data; Results from the survey for these items could be biased to include more respondents who had positive experiences. The questionnaire provided descriptive data about the family but it also required the parent to ‘select’ one child in the family when responding to a series of questions. Thus the complexity of the family health situation was not always captured. There are a number of social paediatrics centres in various stages of development, in several provinces in Canada. Finally, the interview data are limited by the interviewee’s ability to carry out open-ended interviews. Notably in Quebec, where the foundational social pediatric clinics have been established, primary care physicians are used rather than specialists as the point of entry; nurse practitioners are slowly being introduced into Quebec. As such the newer centres align more closely with the PHC structure of the RICHER initiative. Future work examining patients’ experiences with PHC in this model should include these Canadian sites. Strengths of this study are the mixed methods of data collection and analyses to provide insight into the ways in which vulnerability such as poverty and/or being a single-parent household with more than one child can be better understood.

## Conclusion

This study provides beginning evidence that RICHER can better meet the health and health care needs of people, especially those who are vulnerable due to multiple intersecting social determinants of health. Positive interpersonal communication from providers can play a key role in facilitating situations where individuals have an opportunity to experience success in managing their and their family’s health. This success can start a process whereby individuals gain more skills and confidence in managing their health. Finally, this type of PHC delivery model could positively affect health outcomes by organizations and its providers incorporating broad principles of PHC such as community engagement or public participation and intersectoral collaboration, particularly for vulnerable populations.

## Competing interests

None of the authors have any financial or non-financial competing interests.

## Authors’ contributions

SW, JL conceived of the study, participated in its design, administered the survey, and analyzed the data. KK oversaw the day-to-day operations during data collection and analysis. SW, JL, and KK drafted the manuscript. LS and CL facilitated access to the RICHER initiative and participated in the interpretation of the analysis. All authors were involved in analysis of data. All authors read and approved the final manuscript.

## Support

This study was supported by the Canadian Institutes for Health Research and the Michael Smith Foundation for Health Research (Lynam, nominated principal investigator). Dr. Wong was supported by a scholar award from the Michael Smith Foundation for Health Research (CI-SCH-051 (04–1)) and a new investigator award from the Canadian Institutes for Health Research.

## Pre-publication history

The pre-publication history for this paper can be accessed here:

http://www.biomedcentral.com/1471-2431/12/158/prepub
